# Application of IoT in Healthcare: Keys to Implementation of the Sustainable Development Goals

**DOI:** 10.3390/s21072330

**Published:** 2021-03-26

**Authors:** Ángeles Verdejo Espinosa, José Luis López, Francisco Mata Mata, Macarena Espinilla Estevez

**Affiliations:** 1 Electrical Engineering Department, University of Jaén, 23071 Jaén, Spain; 2 Department of Computer Science, University of Jaén, 23071 Jaén, Spain; llopez@ujaen.es (J.L.L.); fmata@ujaen.es (F.M.M.); mestevez@ujaen.es (M.E.E.)

**Keywords:** smart systems, smart grids, IoT, sensors, SDGs, accessibility, social healthcare systems, energy efficiency

## Abstract

We live in complex times in the health, social, political, and energy spheres, and we must be aware of and implement new trends in intelligent social health systems powered by the Internet of Things (IoT). Sustainable development, energy efficiency, and public health are interrelated parameters that can transform a system or an environment for the benefit of people and the planet. The integration of sensors and smart devices should promote energy efficiency and ensure that sustainable development goals are met. This work is carried out according to a mixed approach, with a literature review and an analysis of the impact of the Sustainable Development Goals on the applications of the Internet of Things and smart systems. In the analysis of results, the following questions are answered about these systems and applications: (a) Are IoT applications key to the improvement of people’s health and the environment? (b) Are there research and case studies implemented in cities or territories that demonstrate the effectiveness of IoT applications and their benefits to public health? (c) What sustainable development indicators and objectives can be assessed in the applications and projects analyzed?

## 1. Introduction

Today, technological advances must be accompanied by their applications and implementation in human-inhabited environments, in which public health and energy efficiency play an important role [1,2]. The study of the impact of the Sustainable Development Goals (SDGs) on sensor and Internet of Things (IoT) applications in human environments should be considered essential for the future of our territories [3,4,5]. Energy efficiency and environmental sustainability are intimately linked to people’s health, and the improvement of the quality of human life is today conditioned by technology, sensor networks, intelligent systems, and IoT applications [6].

Smart grids and smart cities should facilitate people’s access to environments, facilitate healthcare services, and promote the safety and happiness in our society. Technologies such as the IoT, the Internet of Energy, artificial intelligence, and the installation of sensors in human environments are used to optimize infrastructures, services, and the strategic planning of communities.

We must design and implement advanced facilities, equipped with sensors and devices that promote the safety and health of people while maintaining a balance between energy use and efficiency. We must train professionals in the technological and engineering sectors, in universities, to keep humans and sustainability at the heart of their designs and projects.

It is essential that the incorporation of intelligent systems is efficient from a technical, safety, and economic point of view. Sensor devices, the Internet of Everything, together with reliable and safe electrical installations and comfortable human environments will lead to cost reduction, higher productivity, and health and safety for people, companies, and institutions.

We cannot forget that the implementation of energy audits in environments inhabited by people is based on a reduction in greenhouse gas emissions, which will have direct effects on the improvement of people’s health, the protection of the environment, and profitability. All these parameters that directly or indirectly affect IoT platforms, energy control, and health should be analyzed as a part of all human health research [7,8,9,10,11,12].

IoT applications and their multiple developments and advances are unstoppable; sensors, intelligent devices, systems, and advanced technological architectures offer great prospects for development in cities and communities in the present and in the near future [13].

The digitization and application of intelligent systems and IoT devices in urbanized environments and in communities inhabited by people is carried out in blocks of analysis, structured in different disciplines, but all of them have a common center of gravity, the trilogy: human–technology–sustainability. We will analyze this trilogy and its main applications, as an introduction to the research carried out [14].

Cities and human-inhabited environments have come under great digital and technological pressure in recent years, and this has been exacerbated by the global pandemic caused by COVID-19 [15,16,17]. The use of intelligent systems, IoT, and advanced engineering systems has played an essential role in the solutions put forward for pandemic management and will continue to gain importance in the future. Institutions at all levels are committed to the energy transition and sustainable development, and we must take them into account in any smart environment developments, research, and implementations [18,19,20].

In the analysis of human environment systems, such as cities or communities, it is necessary to carry out an in-depth study of the conflicts and systems in which an advanced society is involved. These systems and environments must also be involuted as an active part of a whole that brings together, for example, waste management, the measurement of environmental pollution [21,22,23], energy audits, the use of renewable energies, and electrical efficiency [24,25,26,27].

Society needs efficient and healthy resources and everything depends on their management, improved governance, and projects that stimulate the implementation of advanced technological systems to facilitate the path to sustainable development. The management of water, transportation, and agriculture must be taken into account for social growth and human health, since these fields directly involve healthy food and better air quality [5,28,29,30,31,32].

One of the parameters to which we must pay special attention in this study is that of IoT applications for the improvement of people’s health. It will be one of the parameters that we will explore as a common thread throughout the work, in which we will interrelate sustainable development and energy control as necessary elements for its evaluation. Social and public health aspects are at the forefront of the research and work on intelligent systems [33,34,35].

Many intelligent systems applied to human environments will be developed in the next decade, serving to improve the health and quality of life of our societies. They will be ubiquitous, applied in homes, elderly care facilities, hospitals and health centers, schools, and universities, and many institutions will request sensor devices and IoT systems to be implemented for health management [36,37,38].

Governments and political management strategy around intelligent devices, technology, sensors, and IoT systems must be effective and take into account sectors and disciplines such as engineering, computer science, architecture, healthcare, etc. There are multiple cities and territories that are applying the Smart City and Smart Human City strategy to address the future and implement technological advances in cities [39,40,41,42,43].

**Figure 1:**
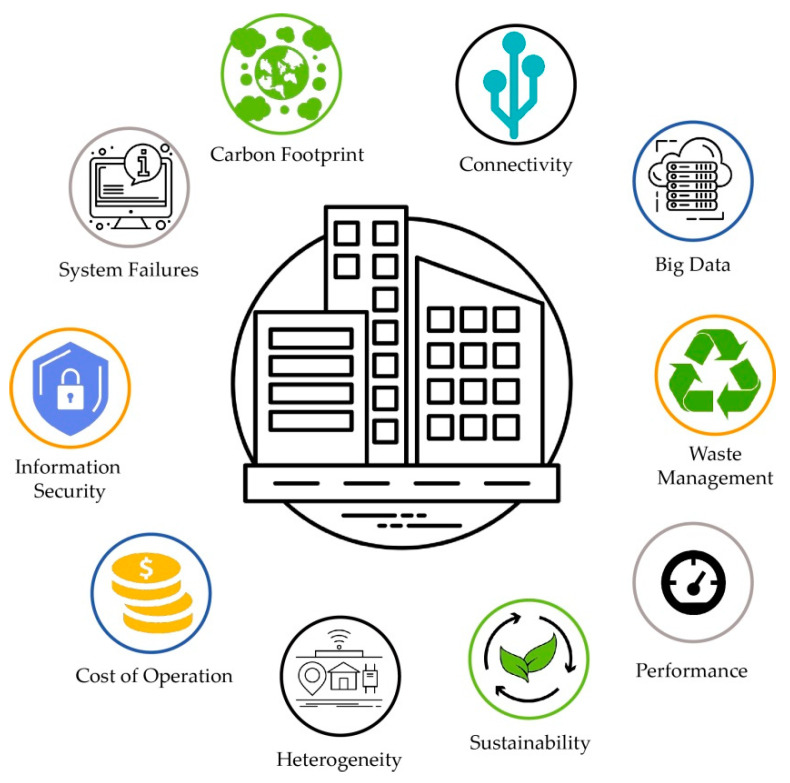
Architectures and components in smart cities [15].

Smart, sustainable, and efficient cities must be designed with sensor systems and IoT applications; the so-called “sensory city” is an integrative model that will enhance people’s lives, management processes, and, with energy efficiency, environmental sustainability and people’s health as the three main pillars [44,45,46,47,48,49]. Advances in the implementation of urban IoT applications and their effectiveness in human environments is unstoppable, so we can indicate some of the most used today. Figure 1 shows some of the research blocks and architectures of present and future smart cities and, within these groups, each of them brings together a large number of sensors, IoT systems, and computer engineering, electrical, electronic, and other technological solutions, which must be multidisciplinary and multifaceted [15].

IoT applications and systems aim to make the Internet even more immersive and integrated into our environment in a natural way. It must be easy to access and interact with any of the devices around us—for example, traffic lights, security cameras, telephones, computers, tablets, vehicles, signs, stores, sensors, health centers, etc. [50,51].

The analysis and location of intelligent systems within urban, rural, and generally human spaces is complex. Sensors, actuators, and devices are located and move with people; they can be fixed but are increasingly mobile—for example, in cell phones or vehicles. However, there are organized architectures and levels of design around how intelligent systems should or can be arranged in human environments, e.g., in cities, communities, or in any other environment [51]. Figure 1 shows one of these architectures and its design.

IoT applications, according to [51], can be analyzed on the basis of the different types of intelligent environments: smart living, smart cities, smart energy, smart transport, smart health, smart industry, smart buildings, smart homes, etc.

In the most common architecture of IoT systems and their applications, it is usually broken down into layers of performance or system implementation. For example, the following main layers [7,15]: application layer, service support/application support layer, network/communication layer, and smart device/sensor layer.

Each of these sub-architectures or working layers integrate the different technologies, devices, sensors, systems, etc., which will be interconnected by means of electrical–electronic and wired or wireless connections. The range of connection elements is very wide and each field of action will have its own specific systems.

Specifically, speaking of systems applicable to people’s health, we can choose and differentiate some of them. In some cases, research is focused on simple, low-cost, ubiquitous devices for monitoring people’s health [52]; in other cases, a general review is made, and different sensors and intelligent systems applied to the care of people at home are detailed [53].

The different existing technologies and systems can be listed, also providing an update and preview of those to come in the future:

1. Technological systems in the smart human home: environmental systems, personal systems, data management systems and interfaces, actuators electrical systems and protections, energy efficiency controllers, software, automation systems, augmented reality, etc.
2. Applications to people’s health: smart healthcare, monitoring of activities of daily living, monitoring for medical management, robotic personal assistance devices, fall monitoring and control devices, telecare for social interaction and leisure, automation for human–machine interaction, implantable medical devices, sensors, microsensors and general-purpose devices, intelligent materials, etc.

**Figure 2:**
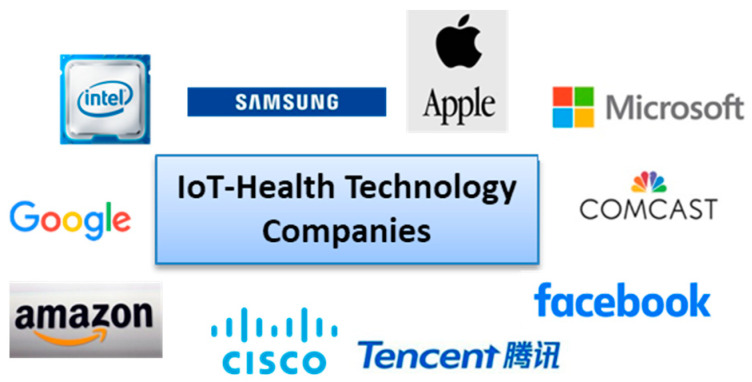
Technology giants in IoT and health.

Innovative projects based on the realization of patents, prototypes, and technological applications using IoT, sensors, and intelligent systems can come from research centers with public or private funding, companies, or public or private institutions, among others. For example, there are large technology companies, such as Telefónica Koa Health [54], which aim to meet the growing demand for digital behavioral health products, or Apple [55], which is committed to the collection and processing of data on mobile devices, through intuitive applications that are close to people and commonly used. Figure 2 shows some of the companies that carry out technological projects applied to people’s health and that are making a major commitment to intelligent social care and healthcare systems empowered by IoT strategy.

The 2030 Agenda of the United Nations (UN) established the Sustainable Development Goals (SDGs) in 2015. This Agenda is an action plan for people, the planet, and prosperity. The 17 goals are comprehensive and indivisible, balancing the three dimensions of sustainable development: economic, social, and environmental [56]. This paper delves into the indicators, goals, and main resources that will be taken into account in the design and implementation of intelligent systems, IoT, and sensors in the framework of the SDGs. Technology, engineering, and the environment are integrated into the research pursuing the advancement of health and sustainable development, for which we will analyze the guidelines governing the SDGs in depth.

It is necessary to propose working methods and protocols to implement the SDGs in smart cities, taking into account citizen participation, governance, energy systems, and technological developments [57]. There are numerous studies, models, and proposals, the most relevant of which we will be analyzed for this work, without forgetting that people’s health is our focal point of action.

There is no doubt that the systems that will make technological change and progress in sustainable development possible will be the IoT, sensors, communications networks, and all the electrical, electronic, computer, and intelligent material devices that will be flexibly integrated into cities and territories. These advances will also bring major energy savings, which are fundamental to the SDG indicators and criteria and essential for energy efficiency and environmental sustainability [58].

The interrelation of IoT systems, health, and energy efficiency has a common nexus that we want to discuss in this paper: the need to design and implement smart technology projects for cities and human environments in which the welfare and quality of life of people coexist in a sustainable and efficient system, which will inevitably lead to substantial benefits for the planet [59]. This paper has also reviewed research on intelligent system designs in smart cities mainly through governance and in collaboration with technology companies, such as Microsoft, Cisco, Amazon, Facebook, Intel, Google, etc. [1,60,61,62].

There must be clarity, transparency, and efficiency in the mass implementation of technological devices, IoT, and sensors, since society may not be prepared for this great change. Fear and ignorance and the digital divide will be important hurdles in the mass installation of systems unknown to a large majority of people. The use of technology for health and energy efficiency must be approached as an unstoppable process that will affect all areas of life. While the general public will become more acquainted with it, their doubts and fears must be addressed.

The pandemic caused by Sars-Cov2 has boosted the research and implementation of useful biosensors and systems capable of obtaining reliable information to prevent, detect, and mitigate the effects of the disease [63], and there is no doubt that various sectors that before were perhaps less prone to the use of technology have now changed their criteria and are willing to use IoT systems, sensors, and other devices in their lives, for example the elderly, who are unaware of the true potential of technology for their welfare.

This work aims to improve knowledge about existing applications and future trends in the implementation of intelligent devices and systems that improve the health of people and communities, from the perspective of energy efficiency and sustainable development. To do this, we will analyze high impact research that is being developed and its effectiveness and whether it really will benefit the health of people and the environment. We will examine its productivity, energy, and economic efficiency and we will address a question that many projects do not take into account: are the sustainable development objectives met in this research?

Our aim is to analyze the technology-health-ODS trilogy and prove that it is effective and will lead to a near future in which sustainability and environmental health are achieved hand in hand with technology. A mixed research approach will review the studies and research offered by the literature on IoT solutions, sensors, smart cities, and smart grids and their importance for public health, energy efficiency, and environmental sustainability. Some of the sources and their implications in different territories will be X-rayed through institutions and technology companies and how they implement the SDG indicators.

The manuscript is structured as follows: Section 1 introduces the subject of our work, Section 2 explains the methodology used, Section 3 analyses the proposed methodological development in depth, details the technological developments of intelligent sensor systems and IoT devices, and their role in health and sustainability, with the fulfilment of the SDGs and their indicators as a common theme. The discussion of the results obtained is detailed in Section 4, and in Section 5, conclusions will be drawn and some proposals and future challenges will be addressed.

## 2. Materials and Methods

**Figure 3:**
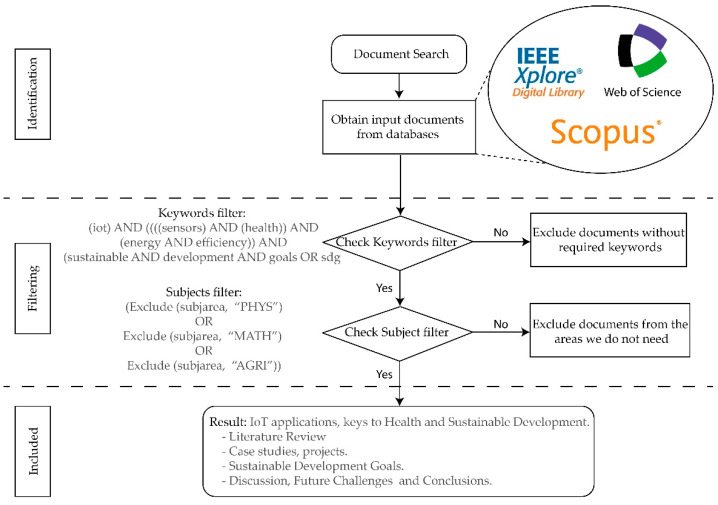
Structure of the methodology.

The work followed a mixed approach [64], in which a literature review, case studies, and real projects provided the material for analysis and implementation of initiatives carried out through IoT systems and applications and smart sensors that improve people’s health. In addition, an improvement of energy efficiency and of environmental sustainability through the SDG proposals was analyzed simultaneously. The choice of a mixed literature review can be beneficial for broad research questions, allowing the various types of evidence to provide a more complete and detailed understanding of the problem by providing multiple perspectives, both quantitative and qualitative, as well as case studies [65,66]. The research was carried out in different phases, which are detailed in Figure 3.

Table 1 shows the sources consulted, which are the scientific databases IEEE Xplore, Web of Science, and Scopus. These are high-impact scientific databases widely used by the scientific–technological community.

Table 2 shows the results obtained according to the search strategies and exclusion filters used.

Following the recommendations of the existing literature in the literature review and case study processes, known as a mixed review [64,65,66,67], we set the objectives of the literature search and the research questions. Our objective was to analyze IoT applications in the field of people’s health and how they improve energy efficiency and comply with the SDGs.

To achieve the objective, we performed a systematic literature review (SLR) in various databases, as set out in Table 1. Subsequently, we posed the research questions and selected the keywords that would form the search strategy in the databases. Afterwards, we analyzed the results obtained and filtered out any areas not related to the stated objectives, finally obtaining a number of references to be used for detailed analysis.

In this work, in addition to an SLR, a mixed method was used, so that in the analysis of the results obtained in the database search, we added research and real case studies implemented by companies or institutions, and we analyzed the most notable of these projects, providing a comprehensive range of knowledge to conduct our review [68].

Table 3 details the research questions we are going to analyze in the paper and the main objectives that respond to these questions.

All the answers to the research questions were analyzed through a qualitative analysis of the documents extracted from the literature search and review, as well as a selection of research of international relevance that, due to its origin or good results, we found interesting for our work.

## 3. Results

In this section, we will analyze the results obtained in the literature search using keywords and filters and also include the selected case studies of the main technology giants, specified in Figure 2. First of all, a quantitative study of the selected manuscripts will be made. This documentation provides an overview of the years in which the research is concentrated, the main areas and sub-areas of study, countries, authors, etc., and offers us a wide range of possibilities for analysis.

### 3.1. Analysis of the Sources Selected in the Literature Review

**Figure 4:**
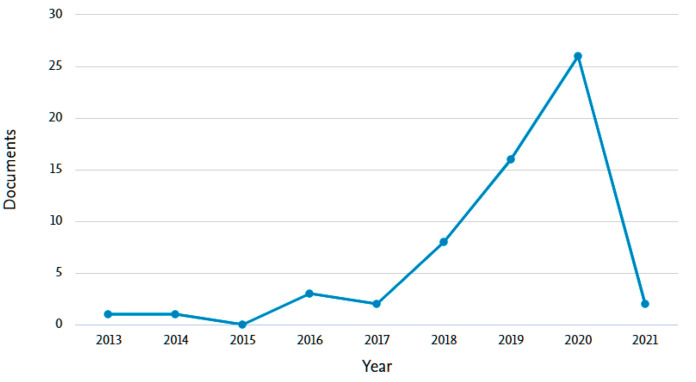
Results by year. Scopus.

Figure 4 provides an overview of the number of sources analyzed and the years of publication of the sources, which shows that the main studies are focused on the years 2019 and 2020.

**Figure 5:**
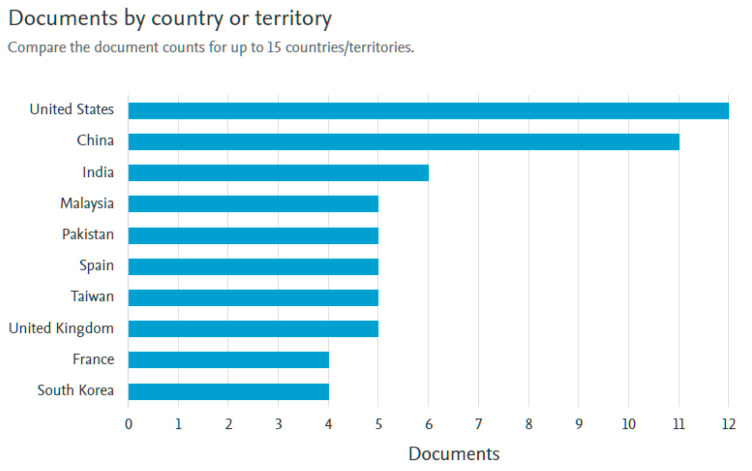
Documents by territory. Scopus.

Figure 5 shows the number of documents published by country of origin of the authors, among which the USA and China stand out.

**Figure 6:**
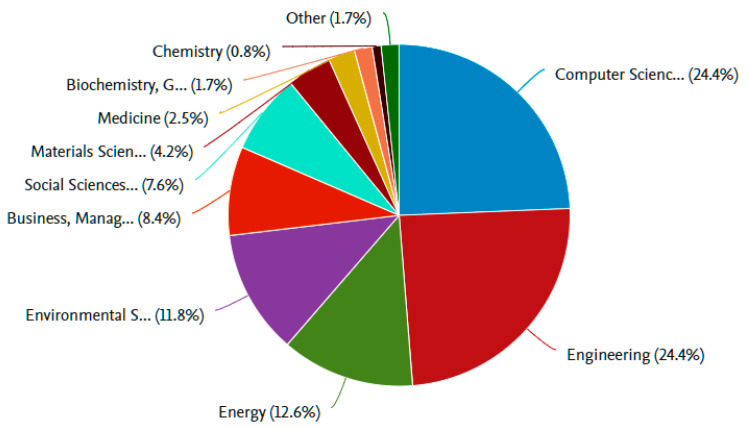
Documents by area. Scopus. Computer Science: 24.9%; Engineering: 24.4%; Energy: 12.6%; Environmental Science: 11.8%; Business, Management and Accounting: 11.8%; Social Sciences: 7.6%; Materials Science: 4.2%; Medicine: 2.5%; Biochemistry, Genetics, and Molecular Biology: 1.5%; Chemistry: 1.7%; Other: 2.9%.

Following the percentages and quantitative parameters, we will begin to evaluate the manuscripts and their main contributions and discussions from a qualitative perspective. We will begin the analysis by following the research questions and examining which of the selected and filtered manuscripts provide answers to the questions. It is interesting to note the main areas of study in the selection of keywords, among which Engineering and Computer Science stand out above the rest, as shown in Figure 6.

Sometimes there are conflicts in the definitions given for smart environments and the systems applied to them and in sustainable environments, when both are complementary and necessary and, in this context, we will address the importance of linking both concepts in all aspects. Sustainability and the SDGs, together with energy efficiency protocols, must be applied to the research of intelligent systems and IoT applications, regardless of the field in which it is carried out. We shall make sure to do so in our case, with our emphasis on health protocols and people’s well-being [2].

Table 4 analyzes the filtered references based on the research questions posed. Each of the sources is analyzed, and a test is carried out to see which area or which specific activity they relate to:

- IoT Applications for Health Improvement;
- Energy Efficiency Indicators analyzed in research;
- Projects or case studies analyzed;
- Sustainable Development Goals.

Among the sources in Table 3, it can be observed that in the 60 references that deal with IoT and its application to health, a small number of them analyze the implications of energy efficiency and the Sustainable Development Goals in their research subject. We will be able to answer the research questions, which will be developed below, according to the quantitative and qualitative analysis carried out previously. Table 4 shows 15 different thematic blocks or groupings of cases and analyses:

1. Block 1: Industry 4.0, health at work, carbon emissions, solid waste management.

There are a large number of research studies that deal with the health of people from the point of view of industry, occupational health, waste management, and, in general, health research from an industrial and occupational perspective. According to Oztemel [25], robots will be dominant in manufacturing, 3D printing, etc., and will dominate the production process. Wearable Internet, big data analysis, sensor-based living, smart city implementations, or similar applications will be the main concern of the scientific community. People’s health will have an ally in the robots and sensors implemented in the industry, which will allow a breakthrough in healthcare and economic and social development [69,70,71,72,73,74,75].

- Analysis and evaluation of energy efficiency at work: Yes.
- Analysis of Sustainable Development Goals: Scarce.

2. Block 2: E-health, elderly adults, environmental resources.

In this block, there are several research studies that focus their analysis on e-health, health, and mobility and focus on the study of elderly people, home care, and disability, among others [77,78,79]. It is a very interesting block of study, especially taking into account the current pandemic and the need for protection and prevention in healthcare for the elderly and people with disabilities. Chiuchisan [76] discusses existing or developing medical devices and technologies that are easy-to-use, affordable, accessible, and sustainable solutions that address a variety of needs, with the goal of ensuring an “active and independent living environment at home” for older adults.

- Analysis and evaluation of energy efficiency at work: Yes.
- Analysis of Sustainable Development Goals: No.

3. Block 3: Cybersecurity, health, and privacy.

Most of the papers explain the problem of security and privacy of people in studies on IoT and sensors applied to health; however, there are some specifically devoted to this issue, such as Hamamreh [80] and Koroniotis [81]. They study the need to design robust, efficient, and versatile security methods for current and future wireless systems.

- Analysis and evaluation of energy efficiency at work: No.
- Analysis of Sustainable Development Goals: No.

4. Block 4: AI, smart cities, governance, urban health, energy efficiency, green IoT.

In this block, different studies have been grouped together, some very similar and others different, but they all have the theme of energy efficiency or sustainability in common, which is why we will group them together for analysis [84,86,87,88].

In the field of Artificial Intelligence (AI), Allam [82] reviews the urban potential of AI and proposes a new framework linking AI technology and cities while ensuring the integration of Smart Cities for the fulfilment of Sustainable Development Goal 11 and the New Urban Agenda. It is the first document to directly address an SDG. Li [83] highlights the cost of using and choosing smart sensors with advanced engineering approaches applied to water quality monitoring management. We must understand that people’s health also depends on water quality applications and energy resources that are an essential part of life.

Tuysuz [85] presents a very interesting concept, “Green IoT”, a novel and powerful element needed to elevate the study of IoT and its applications towards energy sustainability and SDG compliance. It analyses the green perspective of the IoT paradigm and aims to contribute to establishing a global approach to green IoT environments.

- Analysis and evaluation of energy efficiency at work: Yes.
- Analysis of Sustainable Development Goals: Scarce.

5. Block 5: Data mining, health systems, wearable biosensors.

In this set of research, diverse but all related to the IoT and its applications to health and sustainability, we found some works where the subject matter does not really specify the role of energy efficiency at work clearly, nor relate to any of the SDGs. For this reason, the following studies have been grouped into this block: [90,92,94,95,96]. I. Ud Din [89] studies IoT-based smart cities, though not making special reference to energy efficiency or the SDGs. Ana Koren [91] proposes a ZigBee Body Area Network and tests its performance in the context of IoT-based Smart Homes through several simulations with the objective of providing quality solutions in the fields of eHealth. Jin [93] discusses the challenges and opportunities of biosensors as medical devices of the future.

- Analysis and evaluation of energy efficiency at work: No.
- Analysis of Sustainable Development Goals: No.

6. Block 6: Big Data, cyber-physical systems, mobile health, public health.

Rachad Atat [97] presents the taxonomy of cyber-physical systems providing a description of data collection, storage, access, processing, and analysis. Ahrary [99] describes a new approach to designing a production and distribution system for vegetables, using intelligent systems. Konstantellos [98] describes essential theory, recent research, and large-scale use cases that address pressing challenges in cyber-physical system architectures.

- Analysis and evaluation of energy efficiency at work: No.
- Analysis of Sustainable Development Goals: No.

7. Block 7: Smart buildings, healthcare, health monitoring.

In this block, we have grouped research studies that focus on health indoors, in buildings and homes, responding to the needs of people in controlled environments [102,104]. Himeur [92] reviews energy efficiency systems based on data fusion from the consumer’s perspective as a cost-effective strategy for energy reduction in buildings. In terms of health, the main issue at hand, it is concluded that energy efficiency indirectly leads to improvements in terms of the environment and pollution, a benefit to public health. Jia [100] investigates cutting-edge projects and IoT adoptions for the development of smart buildings, both in academic and industrial contexts. In this research work, a detailed literature review of the techniques used for the optimization of energy consumption and scheduling in smart homes has been carried out. 

Detailed discussion has been carried out on different factors contributing to thermal comfort, visual comfort and air quality comfort [101]. Mahmood [103] proposes to reduce energy consumption and understand the impact of home automation on society to achieve the goal of green technology and environmental sustainability. An IoT-based home automation approach integrated with a smart meter is proposed.

- Analysis and evaluation of energy efficiency at work: Yes.
- Analysis of Sustainable Development Goals: Scarce.

8. Block 8: Computing architectures.

We detected in the analysis of the sources that there are some studies on computer architectures [105] that review the potential to improve the performance of current and future IoT communication systems, but it is computationally demanding due to matrix multiplications and inversions. Iddianozie [106] conducts a multidimensional study to address the problem of inferring the semantics of IoT devices using machine learning models, using datasets collected from IoT devices. De [107] discusses the use of fuzzy applications, big data architectures, and the Geographical Information System (GIS) with a higher degree of heuristic and metaheuristic simulations in the agricultural supply chain to develop more robust models.

- Analysis and evaluation of energy efficiency at work: Yes.
- Analysis of Sustainable Development Goals: No.

9. Block 9: Smart city applications architecture, 5G, circular economy.

This block emphasizes the relationship between IoT and its application to health, taking into account the smart city strategy and the circular economy [108,109,111,113,114]. Very interesting sources are included in this block. In [3], the latest advances in IoT technologies, sustainable energy and environment, IoT-enabled smart cities, e-health and ambient assisted living systems, among other topics, are discussed. Although it refers to sustainable systems, it makes no mention of the SDGs—in this sense, it is noteworthy that most sources make little or no reference to the importance of SDG indicators and compliance.

The circular economy in smart cities and related IoT applications is a field of work and analysis that stands out as a novel vision of activity sensorization in business, social, and health applications and many more. However, in the cases studied in this work [110,112], no special mention is made of the SDGs or improving energy efficiency.

- Analysis and evaluation of energy efficiency at work: Yes.
- Analysis of Sustainable Development Goals: No.

10. Block 10: Drones, smart cities, security.

We cannot forget the role that drone technology plays in this era where data and location provide crucial information in all fields of study. In [115], a study of the techniques and applications of collaborative drones and IoT to increase the intelligence of smart cities is presented. These drones and the IoT play a vital role in communication, transportation, agriculture, safety and security, environmental protection, energy saving, weather monitoring, health care, etc.

- Analysis and evaluation of energy efficiency at work: Scarce.
- Analysis of Sustainable Development Goals: No.

11. Block 11: Electronic waste, Sustainable Development Goals (SDGs).

In all the sources analyzed, we found only one in which the involvement of SDGs in IoT research and applications and the impact on health is referred to and studied in depth [116] through an e-waste collection system using IoT.

- Analysis and evaluation of energy efficiency at work: Scarce.
- Analysis of Sustainable Development Goals: Yes.

12. Block 12: Energy management, wireless sensor networks.

Anjum [117] studies RF-based power management to provide power to wireless embedded systems. Dynamic power level maintenance and optimization are analyzed, as well as ensuring reliable communication that adheres to the goal of increased network performance and lifetime [118,119].

- Analysis and evaluation of energy efficiency at work: Yes.
- Analysis of Sustainable Development Goals: No.

13. Block 13: Mobile health applications (m-Health Apps).

This book [120] addresses recent advances from both clinical and technological perspectives to provide a comprehensive view of m-Health. The authors describe the basic concepts of the three scientific technological enablers of m-Health (sensors, informatics, and communications) in detail, and how each of these key ingredients has evolved and matured over the past decade. The book concludes with a detailed discussion of the future of m-Health and presents future directions for shaping and potentially transforming healthcare services in the coming decades. 

Liu [121] applied a new mobile healthcare conceptual framework to develop a measurable evaluation index system with a model for examining consumer adoption of mobile devices.

- Analysis and evaluation of energy efficiency at work: No.
- Analysis of Sustainable Development Goals: No.

14. Block 14: Waste management, logistics.

In this block, we have grouped several research papers related to waste management and logistics, as necessary elements for the preservation of health and closely related to energy and the SDGs. However, we found few references in them to energy efficiency or to any sustainability indicator. In [122], hybrid multi-criteria decision-making methods are used, carrying out a literature review to identify 15 IoT adoption barriers that obstruct IoT implementation in smart cities in India. 

Another work integrated LoRaWAN communication network (network developed using the Internet of Things) with waste sorting equipment to create a system that provides automated garbage can operation, environmental monitoring, and graphical interface monitoring. The system uses electrostatic capacitance-type proximity sensors to determine the types of waste deposited in the trash cans [123].

Regarding logistics, [124] reviews cutting-edge IoT applications in the logistics sector. Although the adoption of IoT potentially generates enormous benefits, there are still barriers that prevent the full adoption of IoT in logistics.

- Analysis and evaluation of energy efficiency at work: Scarce.
- Analysis of Sustainable Development Goals: No.

15. Block 15: Academia.

In this last block, we have reviewed a research paper where the academic field is merged in IoT systems, in which experiences from different educational systems are shared. It discusses digital transformation in academic society and innovative ecosystems in the world [125].

- Analysis and evaluation of energy efficiency at work: No.
- Analysis of Sustainable Development Goals: No.

### 3.2. Analysis of the Sustainable Development Goals and Their Indicators

The Millennium Development Goals (MDGs, 2000–2015) led to improved health and well-being through the collaborative efforts of United Nations (UN) agencies and their constituent Member States. In 2015, the 2030 Agenda for Sustainable Development proposed 17 Sustainable Development Goals (SDGs) [56]. The United Nations Rio+20 summit in Brazil in 2012 committed governments to create a set of SDGs that would be integrated into the follow-up to the Millennium Development Goals (MDGs) after their 2015 deadline [126].

The impact of smart systems on public health is clear, but it is also important to analyze the impact of the SDGs. Therefore, in Table 5, we provide an overview of the relationship between SDGs and their impact on people’s health, taking into account that we are going to study IoT systems and sensor applications in health and healthcare. A high quality healthcare system must take into account the population and their needs, health sector governance, health delivery partnerships, human resources, pharmaceutical laboratories, along with data, IoT, sensor systems, AI, engineering systems, energy efficiency, and advanced technology. Healthcare systems must develop the ability to measure and use data to learn and must be based on four values: they must be for people and they must be equitable, resilient, and efficient [127]. Well-directed political institutions expanding essential public services such as healthcare, education, energy, water, and sanitation are effective in promoting the SDGs [128].

In Table 5, we will analyze the 17 SDGs and the indicators that have the greatest impact on IoT systems applied to health and energy efficiency, necessary for a better quality of life and health of people and, therefore, of their environment.

## 4. Discussion and Future Challenges

In the presented work, a literature review has been carried out taking into account the applications of IoT, its impact on people’s health, and the environment in which we live. Several research questions have been analyzed and examined in the study of the sources investigated, as well as their relationship with the SDGs and their main targets and indicators. We are pleased to observe how a large number of studies and research efforts take into account the SDGs; however, this is generally not done intentionally, as they are not commented on or analyzed in the content of the papers. Therefore, several studies and projects strive to implement IoT designs, installations and applications for people’s health, environmental improvement, environmental protection, and energy efficiency, but not explicitly within the framework provided by the SDGs.

Among the blocks and categories that we have proposed in this work, several can be considered essential to people’s health. They correspond to the SDG indicators that take into account the protection of public health, digital health, autonomous energy systems associated with the maintenance and care of healthcare equipment, and educational and training systems needed to train of healthcare staff in all territories.

Industry, infrastructures, Smart Communities, Smart Human Cities, and, in general, digitalization in all areas will substantially improve people’s health, which inevitably entails the implementation of IoT systems.

Table 6 provides a comparative summary of the SDGs analyzed in depth in the results section, so that we can see more clearly the impact of the Sustainable Development Goals on IoT applications for the improvement of health and the environment, their merits, and demerits.

**Figure 7:**
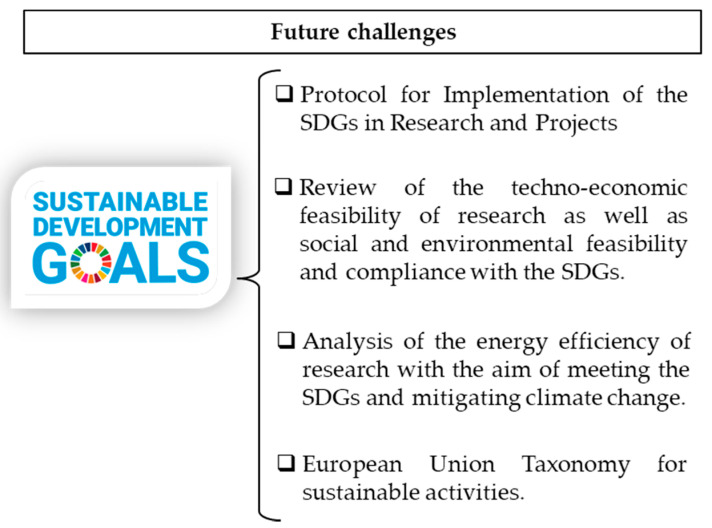
Future challenges.

We have detected some of the challenges to be addressed by our societies and have put forward proposals for future research, which could bring attention to and increase interest in sustainable development and related goals through the SDGs, as a working protocol for research on IoT applications to protect the health of people and their environments, as well as improve energy efficiency. Figure 7 shows a summary of the three proposals or challenges to be addressed in future research on IoT applications to healthcare and compliance with the SDGs.

The first proposal would be the development of a protocol or work methodology in which any research on intelligent systems and IoT applications in all areas and fundamentally in health would pursue a goal or objective within the framework given by the SDGs. For example, if an application is being developed to improve the mobility of the elderly or people with disabilities at home [177], this could be approached according to the indicators that validate compliance with SDG-3-7-9-10-11-16. This validation would be very useful for the purposes of quality assurance in research, implementation by institutions or organizations, etc., as it would prove that the goals and indicators of the SDGs are being met.

The second proposal would be the detection of non-compliance with the SDGs in research; in this sense, any research or work that is subsidized by any government institution, through research projects, grants, contracts, etc., must be shown to comply with any of the SDGs and their main goals, in order to ensure that research of the highest quality and related to sustainable development, efficiency, and protection of people and the environment in line with the SDGs is prioritized. In the engineering research required for an energy installation, the technical–economic feasibility study of the project is no longer enough [173]—it must be sustainable and comply with the SDGs in some of its priority areas.

The third proposal and challenge that is yet to be met, and that we have observed in the analyzed literature, is that many of the research studies pay little or no attention to energy efficiency [97,99], which is essential in any of the fields of study and work promoted by the SDGs. This challenge could be considered in all research areas in a cross-cutting manner, similar to equality or social protection. We cannot promote, design, or implement facilities that do not have energy efficiency as a goal in most of their developments. The SDGs that promote this vector are many, from SDG-1 to SDG-17, and all of them establish energy efficiency as a priority to protect the natural environment, which leads to an improvement in people’s health and entails a mitigation of climate change.

The fourth challenge proposed is to establish the European Union’s strategy on taxonomy for sustainable activities. The EU taxonomy is a classification system that establishes a list of environmentally sustainable economic activities. The EU taxonomy is an enabler that will increase sustainable investment and to implement the European Green Pact. It will provide a tool for businesses, investors, and governance to formulate policies on economic activities that can be considered environmentally sustainable. This system will be a major step towards improving the implementation of IoT applications in all disciplines and primarily in energy and health [230].

For all the above, from what has been analyzed in the literature review, both from a quantitative and qualitative point of view and in the process of reviewing the SDGs and energy efficiency parameters, we consider that these three proposals could considerably improve future research and work in the field of sustainability and drive progress in the health, social, economic, and environmental spheres.

For the implementation of all projects and work in less developed countries through low-cost means, renewable energy systems should be a further objective and indicator, to be considered essential in the research on IoT applications in all fields, especially in public health.

## 5. Conclusions

The deployment of IoT applications and sensors in our lives is inevitable, and every day there are more connected devices and a greater number of systems that require the implementation of these technologies, especially to improve quality of life, health, safety, and in many other applications. Technology in general and the interconnectedness of different elements integrated in our homes, at work, or in the environment, parks and gardens, street furniture, traffic lights, vehicles, etc., are part of our daily lives and are increasingly essential. IoT technology and applications must be low cost and accessible to a large number of people and environments, so that the implementation of devices and their benefits to society will be enhance equity and, ultimately, will be unstoppable. The tools needed to improve the health of people and their environments, as we have analyzed in this work, are multiple and have no limit: we are developing more and more novel and original systems that are adaptable to any situation and any territory.

The fact of being able to demonstrate that IoT applications benefit public health and sustainability by undergoing verification according to the indicators and goals of the SDGs would represent progress in our commitment to integrate technology in all corners of the planet and achieve universal impact, without distinction between countries, races, environments, or populations.

At the beginning of this article, we proposed a bibliographic review to examine the research on IoT systems and devices, their applications and their benefit to public health and sustainability. To this end, we posed several research questions, which we have analyzed in the various sections of the work. On this basis, we conclude that there are multiple and very different works, sensor devices, and intelligent systems aimed at promoting and protecting people’s health and, in turn, the territories and the environment they inhabit. Therefore, by including a more detailed analysis of the SDGs and the implications of their goals and objectives on these devices, in Table 5, we have validated that there are many works and studies where, despite not explicitly including references to the SDGs in their research, many of their indicators are met and verified. 

Artificial intelligence and its applications in the IoT and integration in society will be essential for the fulfilment of a large part of the SDGs, so it must be integrated extensively. Meanwhile, awareness should be raised on all of the social, health, and environmental benefits that the implementation of AI projects and developments will entail. There are studies and reports that endorse this, such as [231,232].

To conclude, some of the highlights we have observed are: (a) the proposed analysis offers us filtered information on the research on IoT systems and applications in the field of public health and their benefits to sustainability; (b) to achieve a comprehensive social and institutional system for the protection of public health in different communities and territories, it is necessary to innovate and continue research on sensor technologies and IoT applications, since these offer information, data management, and continuous improvement of healthcare solutions, often at low cost and deployable within people’s family circles and homes; (c) the data obtained from the multiple applications implemented will allow greater control of the security of the population, for the improvement of society and its territories, which will lead to an improvement in institutional management and governance; (d) security will be important and therefore should be taken into account for the management of intelligent systems and applications geared at improving public health and efficiency; (e) the SDGs should be a vehicle for IoT development, offering its goals and objectives as a framework for the design, installation, and management of the technology to be implemented.

## Figures and Tables

**Table 1:** Consulted databases.

Source	URL
IEEE Xplore	http://ieeexplore.ieee.org (accessed on 18 November 2020).
Web Of Science (WOS)	https://clarivate.com/webofsciencegroup (accessed on 19 November 2020).
Scopus	http://www.scopus.com (accessed on 20 November 2020).

**Table 2:** Methodological search strategy and filters.

Search for Keywords	Results
(TITLE-ABS-KEY (IoT) and (Sensors) and (Health) and (Energy and Efficiency) and (Sustainable and Development) and (Goals) or (SDG)).	78
**Filters applied**	
**Filter:** Exclusion of some subject areas: (Exclude (Subj_area, “Physics and Astronomy”) or Exclude (Subj_area, “Mathematics”) or Exclude (Subj_area, “Agricultural and Biological Sciences”)).	18
**Total Results (78–18)**	**60**

**Table 3:** Identified research questions and their objectives.

Q. N°	Identified Research Questions	Objectives
RQ.1.	Are IoT applications key to the improvement of people’s health and the environment?	The objective is to demonstrate through literature and evaluation of SDGs the importance of IoT applications for health and environment
RQ.2.	Are there research and case studies implemented in cities or territories that demonstrate the effectiveness of IoT applications and their benefits to public health?	The objective is to analyze through a literature review those studies and research based on IoT applications that implement or design systems that improve people’s health and sustainability.
RQ.3.	What sustainable development indicators and objectives can be assessed in the applications and projects analyzed	The objective is to analyze all SDGs and their indicators in order to locate those that are directly involved in the research and implementation of IoT applications.

**Table 4:** Analysis of the sources consulted and their subject matter.

Research Focused on IoT Applied to:		Analyze Energy Efficiency	Analyze the SDGs	Source
1	Industry 4.0, health at work, carbon emissions, solid waste management	X	o	[25,69,70,71,72,73,74,75]
2	E-health, elderly adults, environmental resources	X	-	[76,77,78,79]
3	Cybersecurity, health and privacy	-	-	[80,81]
4	IA, smart cities, governance, urban health, energy efficiency, green IoT.	X	o	[82,83,84,85,86,87,88]
5	Data mining, health systems, wearable biosensors	-	-	[89,90,91,92,93,94,95,96]
6	Big Data, cyber-physical systems, mobile health, public health	-	-	[97,98,99]
7	Smart buildings, healthcare, health monitoring	X	o	[92,100,101,102,103,104]
8	Computing architectures	X	-	[105,106,107]
9	Smart city applications architecture, 5G, circular economy	X	-	[3,108,109,110,111,112,113,114]
10	Drones, smart cities, security	o	-	[115]
11	Electronic waste, Sustainable Development Goals (SDGs)	X	X	[116]
12	Energy management, wireless sensor networks	X	-	[117,118,119]
13	Mobile health applications (m-Health Apps)	-	-	[120,121]
14	Waste management, logistics Smart cities	o	-	[122,123,124]
15	Academia	-	-	[125]

(X): Yes; (o): Escarce; (-): No.

**Table 5:** Impact of the Sustainable Development Goals on IoT applications for improving health and the environment.

SDGs	Targets Goals and Application to IoT Systems
**Goal 1: End poverty in all its forms everywhere.** 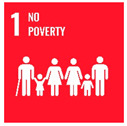 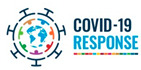	Targets 1.1–4, 1.a.: Implement appropriate social protection measures for all. Ensure that all men and women have equal rights to economic resources, as well as access to basic services, ownership and control of land, inheritance, natural resources, and technology. To support the poorest and most vulnerable, the UN has issued a Framework for the immediate socio-economic response to COVID-19, calling for an extraordinary scale-up of international support and political commitment to ensure that people everywhere have access to essential services and social protection. IoT Application Solutions [89,129,130,131,132,133]: Social protection is achieved through advanced healthcare systems. Coverage will be achieved through the design of IoT systems and technological applications that can be applied to different territories. Energy efficiency will be led by advances in renewables and efficient energy applications for powering smart devices. Natural resources will benefit from the introduction of low-cost technologies, sensors, and efficient applications. Economic, social, and human resources will be essential for the progression and implementation of intelligent systems, sensors, and applications that achieve real progress in vulnerable populations. These resources must provide the means to make progress in bridging the technology gap as well as the health and energy gaps.
**Goal 2: Zero hunger.** 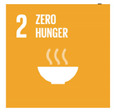 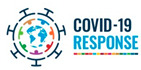	Targets 2.1–4, 2.a.: Doubling the agricultural productivity and incomes of small-scale food producers, particularly women, indigenous peoples, family farmers, pastoralists, and fisherfolk, through secure and equitable access to land, knowledge, markets, and opportunities for value addition and off-farm employment generation. Ensure the sustainability of food production systems and implement resilient agricultural practices that increase productivity and production, contribute to the maintenance of ecosystems, strengthen resilience to climate change, extreme weather events, droughts, floods, and other disasters, and progressively improve soil and land quality. Increase investments in rural infrastructure, agricultural research, and extension services, technological development, and plant and livestock gene banks. In light of the pandemic’s COVID-19 effects on the food and agricultural sector, prompt measures are needed to ensure that food supply chains are kept alive to mitigate the risk of large shocks that have a considerable impact on everybody, especially on the poor and the most vulnerable. IoT Application Solutions [3,134,135,136,137,138]: Agricultural productivity and improved nutrition can be achieved with the planning, design, and implementation of intelligent IoT systems and sensors applied to water management, pest control, etc. The generation of opportunities and added value in sales and marketing of agricultural products will see in technology and smart systems a great opportunity for improvement. Technological systems and IoT applications, irrigation sensors, etc., will be necessary to improve sustainability in food production systems. This, in turn, will lead to improvements in public health. The control of meteorological disasters can be mitigated with drone systems with devices for tracking and locating people and transferring them to healthcare centers. Investments in renewable energy production facilities, which will provide clean energy and energy efficiency to isolated communities and agricultural and healthcare infrastructures, will play an important role in this goal.
**Goal 3: Ensure healthy lives and promote well-being for all at all ages.** 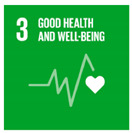 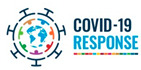	Targets 3.1–3.9, 3.a–d: Ensure healthy lives and promote well-being for all at all ages. Reduce the global maternal mortality ratio. End preventable deaths of newborns and children under five years of age. End the epidemics of AIDS, tuberculosis, malaria, and neglected tropical diseases and combat hepatitis and waterborne and other diseases. Reduce by half the number of deaths and injuries caused by road traffic accidents worldwide. Ensure universal access to sexual and reproductive health services. Achieve universal health coverage and access to quality health services, safe, effective, and affordable medicines and vaccines. Reduce the number of deaths and illnesses caused by hazardous chemicals and air, water, and soil pollution. Support research and development of vaccines and medicines and facilitate access to affordable essential medicines and vaccines in accordance with the Doha Declaration on the TRIPS Agreement and Public Health, which affirms the right of developing countries to make maximum use of the provisions of the Agreement on Trade-Related Aspects of Intellectual Property Rights regarding flexibilities to protect public health. Increase health financing and the recruitment, development, training, and retention of the health workforce in developing countries. Strengthen the capacity of all countries in early warning, risk reduction, and health risk management. The World Health Organization (WHO) has been leading the global effort to tackle COVID-19. The Strategic Preparedness and Response Plan, produced by WHO and partners, outlines the public health measures that countries should take to prepare for and respond to COVID-19. The Strategy Update of April 2020 provides further guidance for the public health response to COVID-19 at national and subnational levels, and highlights the coordinated support that is required from the international community to meet the challenge of COVID-19. IoT Application Solutions [77,78,139,140,141,142,143,144,145,146,147]: Intelligent systems, sensors, IoT, technologically advanced medical devices, communications networks, etc., represent a significant improvement in the synergies between medical centers in vulnerable countries and advanced healthcare centers in developed countries. In recent months, it has been proven that technological systems and IoT applications are of great importance in the research, application, and prevention of diseases and pandemics worldwide, when it comes to vaccine and drug manufacturing, logistics, population vaccination planning and management, data management, and patient monitoring at home, among other efforts. Traffic accident prevention and control systems have an ally in technological systems and advanced IoT applications, for example, in user applications that inform and warn of vehicle congestion in cities and highways, accident warnings, alarm systems in adverse weather situations, among others. Development of efficient and high-quality vaccines, where sensor applications, IoT, and intelligent systems play a great role. Technology, in all its fields, has a fundamental mission for the effective development and distribution of vaccines to the entire population, with applications to data management, including patient data, vaccination control, health and safety, among others. In the area of air and soil pollution and the detection of chemical elements in the environment, there are multiple applications of sensors and intelligent systems that detect, transmit, manage, and prevent such problems. For example, there are sensors for humidity, toxins in agriculture, atmospheric pollution, etc., which represent a significant advance in this sector. Technology and its ramifications bring unquestionable value to the protection of health and to the development of global healthcare. Until just a few years ago, it was unthinkable that an operation or a medical diagnosis could be carried out by videoconference, using computer, electrical, and telecommunications technology. Today this is possible, and the accessibility of healthcare has given hope to many isolated territories without access to the most basic health care. Remote training of healthcare staff is now also possible thousands of kms away, thanks to computer technologies, telecommunications and renewable energy systems, such as solar, wind, or mini-hydraulic energy. Risk reduction and global healthcare management will be fully effective and accessible if smart systems are integrated as a common thread, supported by IoT technologies and local energy management.
**Goal 4: Quality education** 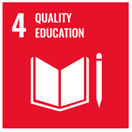 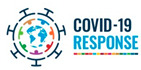	Targets 4.1–4.7: Ensure inclusive, equitable, and quality education and promote lifelong learning opportunities for all. Ensure: Free, quality primary and secondary education. Quality early childhood and preschool care and development. Equal access for men and women to quality technical, vocational, and higher education, including university education. Skills for youth and adults for access to employment, decent work, and entrepreneurship. Elimination of gender disparities in education. Equal access to all levels of education and vocational training for vulnerable people, people with disabilities, and indigenous peoples. Ensuring literacy and numeracy for the majority of the world’s population. Ensure training to promote sustainable development, human rights, gender equality, the promotion of a culture of peace and non-violence, global citizenship and the appreciation of cultural diversity and the contribution of culture to sustainable development. To protect the well-being of children and ensure they have access to continued learning, UNESCO in March 2020 launched the COVID-19 Global Education Coalition, a multi-sector partnership between the UN family, civil society organizations, media, and IT partners to design and deploy innovative solutions. Together they help countries tackle content and connectivity gaps, and facilitate inclusive learning opportunities for children and youth during this period of sudden and unprecedented educational disruption IoT Application Solutions [148,149,150,151,152]: All applications of intelligent systems, their variants, along with the design and implementation of sensors and IoT, can contribute to improving the quality of basic education, as well as training in fields such as science, technology, and mathematics, for entire populations and in any environment in the world. While it is true that in many territories and areas that are especially vulnerable, with armed conflicts, isolated and/or in indigenous and rural areas, energy and basic resources are needed to access quality education, but the technology exists, can be implemented, and could provide a large part of the world’s population with distance learning, requiring minimal technological and human resources. The training of teachers for the work of improving literacy and quality education requires a large technological network that is affordable and accessible in developing countries, which could be supplied by human resources existing in developed countries, through development cooperation and through advanced and free technological platforms.
**Goal 5: Achieve gender equality and empower all women and girls.** 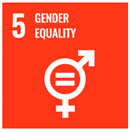 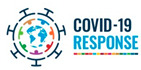	Targets 5.1–5.4, 5.a–b: End all forms of discrimination against all women and girls worldwide. Eliminate all forms of violence against all women and girls. Recognize and value care and unpaid domestic work. Ensure women’s participation in political, economic, etc., life. Improve the use of enabling technology, in particular information and communications technology, to promote women’s empowerment. Every COVID-19 response plan, and every recovery package and budgeting of resources, needs to address the gender impacts of this pandemic. This means: (1) including women and women’s organizations in COVID-19 response planning and decision-making; (2) transforming the inequities of unpaid care work into a new, inclusive care economy that works for everyone; and (3) designing socio-economic plans with an intentional focus on the lives and futures of women and girls. IoT Application Solutions [120,153,154,155]: Technological systems and IoT devices and applications are making a breakthrough in gender equality education and in the prevention, eradication, and monitoring of violence against women and girls. We can carry out educational training through intelligent platforms, control abusers through electro-computer devices for the safety of the victims, as well as empower and raise awareness about the work and participation of women in all areas of life. The use of technology as an education and empowerment tool for women is one of the goals of objective 5, which is justified by the need to acquire scientific–technological knowledge in order to acquire economic and social independence and achieve personal and social progress for women and girls.
**Goal 6: Ensure access to water and sanitation for all.** 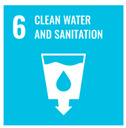 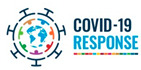	Targets 6.1–6.6: Achieve universal and equitable access to safe and affordable drinking water for all. Achieve access to adequate sanitation and hygiene. Improve water quality, eliminate dumping, increase recycling. Increase water use efficiency and ensure sustainable freshwater withdrawal and supply to address water scarcity. Implement integrated water resource management. Protect and restore water-related ecosystems, including mountains, forests, wetlands, rivers, aquifers, and lakes. Availability and access to water, sanitation, and hygiene (WASH) services is fundamental to fighting the virus and preserving the health and well-being of millions. COVID-19 will not be stopped without access to safe water for people living in vulnerability, UN experts said. IoT Application Solutions [116,156,157,158]: IoT systems and sensors used in water management and their benefits for the population are multiple. The health of people and the natural environment depend on the optimal management of water, its recycling, its quality, and access to it for all people. There are technologies that use IoT applications for this purpose to control water quality through humidity and pollution sensors, renewable energy systems that extract and purify water efficiently from wells, and aquifers for use by the population and advanced technological systems for water purification, among others.
**Goal 7: Ensure access to affordable, reliable, sustainable, and modern energy.** 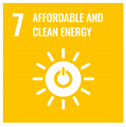 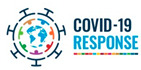	Targets 7.1–7.4, 7.a–b: Ensure universal access to affordable, reliable, and modern energy services. Increase the share of renewable energy. Double the global rate of energy efficiency improvement. Improve international cooperation to facilitate access to clean energy research and technology. Promote investment in energy infrastructure and clean energy technology. Expand infrastructure and improve technology for the provision of modern, sustainable energy services for all in developing countries. Lack of access to energy may hamper efforts to contain COVID-19 across many parts of the world. Energy services are key to preventing disease and fighting pandemics—from powering healthcare facilities and supplying clean water for essential hygiene, to enabling communications and IT services that connect people while maintaining social distancing. IoT Application Solutions [159,160,161,162,163,164,165,166,167]: Solutions with IoT applications in the energy field are an essential and necessary to improve public health worldwide. Renewable energy installations, solar thermal, solar photovoltaic, wind, hydro, geothermal, marine, etc., are important to achieve high-impact technological and scientific progress. People, communities, and the environment will directly benefit from the implementation of smart systems and IoT solutions applied to the energy sector geared towards efficiency and sustainability. For example, installations of solar photovoltaic solutions in schools, health centers, and rural populations in developing countries have made a significant difference to the population and directly improved public health and education systems. In any country or community, energy projects and installations of any type and in any field cannot be conceived without a remote management system, telemetry, remote fault control, installation of temperature, climate and pressure sensors, among others, in addition to instantaneous data transmission to monitor and protect the installations, artificial intelligence, environmental intelligence, or precision technology.
**Goal 8: Promote inclusive and sustainable economic growth, employment, and decent work for all.** 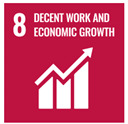 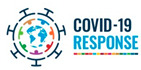	Targets 8.1–8.10, 8. a–b: Maintain per capita economic growth in accordance with national circumstances and, in particular, gross domestic product growth of at least 7% per year in the least developed countries. Achieve higher levels of economic productivity through diversification, technological upgrading, and innovation. Promote development-oriented policies that support productive activities, decent job creation, entrepreneurship, creativity, and innovation. Improve overall resource efficiency in consumption and production. Achieve full and productive employment and decent work for all women and men, including youth and persons with disabilities, and equal pay for work of equal value. Take measures to eradicate forced labor and end modern slavery, child labor, and the use of child soldiers. Protect labor rights and safe working environments. Design and implement policies to promote sustainable tourism that creates jobs and promotes local culture and products. Strengthen the capacity of financial institutions. Increase Aid for Trade support to developing countries. Develop and implement the International Labor Organization’s Global Jobs Pact. The COVID-19 pandemic has caused a historic recession with record levels of deprivation and unemployment, creating an unprecedented human crisis that is hitting the poorest hardest. In April 2020, the United Nations released a framework for the immediate socio-economic response to COVID-19, as a roadmap to support countries’ path to social and economic recovery. IoT Application Solutions [25,131,168,169,170,171]: The promotion of a modern, sustainable economy that protects workers requires the consolidation and implementation of first-level technological systems, such as IoT applications, sensors, and intelligent systems that can generate knowledge and knowledge transfer in the economic and commercial spheres and for effective work. The immersion of devices that facilitate work in rural areas, in less developed countries, in remote or difficult-to-access environments and locations will be essential to achieve progress in the jobs and companies that handle the maintenance of populations in industrial deterioration. The commitment to technology and management through IoT devices is and will be the best economic and entrepreneurial structure for many people and new or existing companies.
**Goal 9: Build resilient infrastructure, promote sustainable industrialization, and foster innovation.** 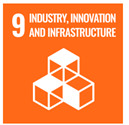 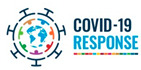	Targets 9.1–9.5, 9.a–c: Develop quality, reliable, sustainable, and resilient infrastructure to support economic development and human well-being, with a focus on affordable and equitable access for all. Promote inclusive and sustainable industrialization. Increase the access of small industrial enterprises to financial services, including affordable credit. Improve infrastructure and modernize industries to make them sustainable, with greater adoption of technologies. Improve scientific research and encourage innovation. Facilitate sustainable infrastructure development through increased financial, technological and technical support to African and other least developed countries. Support national technology development, research, and innovation in developing countries. Increase access to information and communications technology and strive to provide universal and affordable access to the Internet in the least developed countries. IoT Application Solutions [69,131,172,173,174,175,176,177]: This objective aims to promote infrastructures, industrialization, and scientific research to enhance the business sector, drive environmental sustainability, and encourage innovation. IoT technologies and systems play a very important role, where modern industrialization, in all its variants, is undergoing an adaptation to high-level productive technological processes, to be able to compete and protect the environment. Any production process that takes care of people, protects their health, and drives environmental sustainability requires an implementation plan integrating intelligent systems, IoT sensor technologies, energy efficiency, and renewable and sustainable resources.
**Goal 10: Reduce inequality within and among countries.** 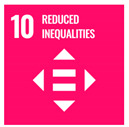 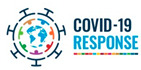	Targets 10.1–10.7, 10. a–c: Progressively achieve and maintain income growth for the poorest population. Empower and promote the social, economic, and political inclusion of all people. Guarantee equal opportunities, eliminate discriminatory laws, policies, and practices, and promote appropriate laws, policies, and measures. Adopt fiscal, wage, and social protection policies. Improve the regulation and monitoring of global financial institutions and markets and strengthen the implementation of such regulations. Ensure greater representation of developing countries in decision-making in international economic and financial institutions. Facilitate orderly, safe, regular, and responsible migration and mobility of people with planned and well-managed migration policies. Encourage official development assistance and financial flows, including foreign direct investment, to states where the need is greatest. To ensure that people everywhere have access to essential services and social protection, the UN has called for an extraordinary scale-up of international support and political commitment, including funding through the UN COVID-19 Response and Recovery Fund, which aims to support low- and middle-income countries and vulnerable groups who are disproportionately bearing the socio-economic impacts of the pandemic. IoT Application Solutions [131,171,178,179,180,181,182,183,184,185]: There is no doubt that technology and its applications in all areas of society represent significant and essential progress. Under this objective, more egalitarian growth would lead the most vulnerable countries with the greatest needs in terms of entrepreneurship, business, health, education, and in general, in all areas, to gradually catch up with the most developed countries. Societies will be empowered through training and the implementation of technological systems and their applications in companies, local businesses, e-commerce, etc. Likewise, regulated migration, border control, and the management of population movements can be carried out with intelligent systems and IoT and data management applications.
**Goal 11: Make cities inclusive, safe, resilient, and sustainable.** 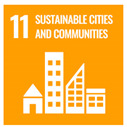 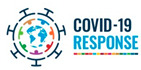	Targets 11.1–11.7, 11.a–c: Ensure access to adequate, safe, and affordable housing and basic services. Provide access to safe, affordable, accessible, and sustainable transport systems, improving road safety, with special attention to the needs of people in vulnerable situations, women, children, people with disabilities, and the elderly. Improve inclusive and sustainable urbanization and capacity for participatory, integrated and sustainable planning, and management of human settlements. Protect and safeguard the world’s cultural and natural heritage. Reduce deaths and the number of people affected by disasters. Reduce the environmental impact of cities, including special attention to air quality and municipal and other waste management. Provide universal access to safe, inclusive, and accessible green and public spaces, particularly for women and children, the elderly, and people with disabilities. Support positive economic, social, and environmental linkages between urban, peri-urban, and rural areas. Increase the number of cities and human settlements that adopt and implement integrated policies and plans towards inclusiveness, resource efficiency, climate change mitigation and adaptation, and disaster resilience. Support least developed countries, including through financial and technical assistance, in the construction of sustainable and resilient buildings using local materials. UN-Habitat, the UN agency for housing and urban development, is working with national and local governments to help them prepare for, prevent, respond to, and recover from the COVID-19 pandemic. IoT Application Solutions [15,82,131,186,187,188,189,190,191,192,193,194,195]: Cities and human communities must protect the environment we are a part of. The environment, in which we coexist with animals, plants, and natural resources necessary for life, is essential for human existence, development, progress, and quality of life. Smart cities, sustainable cities, and smart grid strategies must come together to form smart human communities. The use of IoT systems and applications will help us with the planning, design, and improvement of any projects integrated in communities to make them more sustainable and efficient. For example, having reliable and updated data on urban mobility, pollution due to transport, or weather will enable prevention and action at an institutional or individual level. Providing smart cities or smart communities with sensors and IoT devices will give them greater control of the environment; improve urbanization, accessibility, transportation, water and air quality, and waste control; and prevent natural disasters or mitigate them with updated information and data management.
**Goal 12: Ensure sustainable consumption and production patterns.** 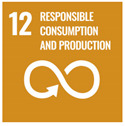 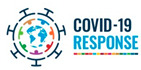	Targets 12.1–12.8, 12.a–c: Implement programs on sustainable consumption and production. Achieve sustainable management and efficient use of natural resources. Reduce global food waste. Achieve environmentally sound management of chemicals and all wastes throughout their life cycle and reduce their release into the air, water, and soil to minimize their adverse impacts on human health and the environment. Reduce waste generation through prevention, reduction, recycling, and reuse. Encourage companies to adopt sustainable practices and integrate sustainability information into their reporting cycle. Promote sustainable procurement practices. Ensure that people have the information and awareness relevant to sustainable development and lifestyles in harmony with nature. Support developing countries to strengthen their scientific and techno-logical capacity to move toward more sustainable consumption and production patterns. Develop and implement tools to monitor the impacts of sustainable development for sustainable tourism that creates jobs and promotes local culture and products. The emergence of COVID-19 has underscored the relationship between people and nature and revealed the fundamental tenets of the trade-off we consistently face: humans have unlimited needs, but the planet has limited capacity to satisfy them. We must try to understand and appreciate the limits to which humans can push nature, before the impact is negative. Those limits must be reflected in our consumption and production patterns. IoT Application Solutions [131,196,197,198,199,200,201,202,203]: The solutions for responsible production and consumption must necessarily include the search for technological applications and solutions that facilitate data management, pollution, and waste control and analysis of the production chain and life cycle of products. Human health goes hand in hand with healthy food, proper management of natural resources, and adequate water and air quality, which must be controlled and monitored, both in communities in developed countries and in poorer ones. Monitoring through sensors and smart devices involves the implementation of IoT technologies, which, when deployed in the production and waste chains, will be able to report and provide timely data to act on anomalies in life cycles. For example, sensors are already being used to monitor urban waste, through containers, by monitoring schedules and with container filling data management. Those who do not comply with proper recycling regulations can then be penalized. There are multiple communication and information devices for the promotion of local products, rural tourism, and the commercialization of local food, which are being disseminated through technological platforms, internet, and other means. Additionally, crops and livestock are controlled through IoT systems for improved food management.
**Goal 13: Take urgent action to combat climate change and its impacts.** 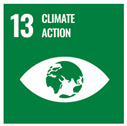 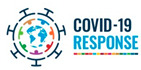	Targets 13.1–2, 13.a–b.: Strengthen resilience and adaptive capacity to climate-related hazards and natural disasters in all countries. Strengthen resilience and adaptive capacity to climate-related hazards and natural disasters in all countries. Integrate climate change measures into national policies, strategies, and planning. Improve education, awareness, and human and institutional capacity on climate change mitigation, adaptation, impact reduction, and early warning. Fulfill the commitment made by developed countries in the United Nations Framework Convention on Climate Change. The United Nations Framework Convention on Climate Change is the main international and intergovernmental forum for negotiating the global response to climate change. As countries move toward rebuilding their economies after COVID-19, recovery plans can shape the 21st century economy in ways that are clean, green, healthy, safe, and more resilient. The current crisis is an opportunity for a profound, systemic shift to a more sustainable economy that works for both people and the planet. IoT Application Solutions [86,166,204,205,206,207,208,209]: Action against climate change is one of the most important impacts that we must analyze from the technological and scientific point of view. The mitigation of specific pollutants that cause climate change, such as carbon dioxide (CO_2_) emissions, is essential so that in the coming years we can move forward and obtain satisfactory results in this fight. IoT systems and applications can and are used in the fight against climate change in a cross-cutting manner and on many fronts, such as the control, measurement, management, and reporting of data on pollutant emissions in the industrial and transportation sectors, or through sensors installed in cities and potentially hazardous environments.
**Goal 14: Conserve and sustainably use the oceans, seas, and marine resources.** 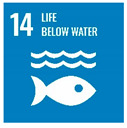 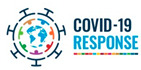	Targets 14.1–7, 14.a–c.: Prevent and reduce marine pollution, particularly from land-based activities, including marine debris and nutrient pollution. Sustainably manage and protect marine and coastal ecosystems. Minimize and address the impacts of ocean acidification. Regulate catch and end overfishing, illegal fishing, and destructive fishing practices and implement science-based management plans. Conserve at least 10% of coastal and marine areas, in accordance with national and international law and based on the best available scientific information. Increase scientific knowledge, develop research capacity, and transfer marine technology, taking into account the Criteria and Guidelines of the Intergovernmental Oceanographic Commission on the Transfer of Marine Technology. Provide small-scale artisanal fishers with access to marine resources and markets. Improve the conservation and sustainable use of the oceans and their resources through the implementation of international law as reflected in the United Nations Convention on the Law of the Sea. The health of the ocean is intimately tied to our health. According to UNESCO, the ocean can be an ally against COVID-19: Bacteria found in the depths of the ocean are used to carry out rapid testing to detect the presence of COVID-19, and the diversity of species found in the ocean offers great promise for pharmaceuticals. IoT Application Solutions [210,211,212,213]: The health of people and our environment depends on the health of our oceans. Marine waste and dumping is a major international problem that is having a serious impact on the oceans and smaller seas and must be managed accordingly. The control of ocean pollution can be improved through intelligent applications and systems using IoT, sensors, among other devices, since they can help prevent ocean dumping and provide data for the control and improvement of the marine waste problem and its consequences. For example, there are devices that measure ocean acidification, changes in color, size, and density of marine species, algae, plants, etc., that can provide significant data for the control of sea water quality. In addition, ships can be equipped with intelligent devices that provide a large amount of high-quality data, providing information that is crucial for marine management.
**Goal 15: Sustainably manage forests, combat desertification, halt and reverse land degradation, and halt biodiversity loss.** 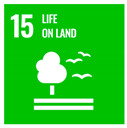 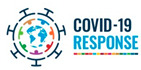	Targets 15.1–9, 15.a–c: Ensure the conservation, restoration, and sustainable use of terrestrial and inland freshwater ecosystems and their services, in particular forests, wetlands, mountains, and drylands. Promote the implementation of sustainable management of all types of forests, stop deforestation, restore degraded forests. Combat desertification and restore degraded land and soil. Ensure the conservation of mountain ecosystems, including their biodiversity. Take urgent and significant measures to reduce the degradation of natural habitats, halt the loss of biodiversity, and protect and prevent the extinction of threatened species. Promote the fair and equitable sharing of benefits derived from the use of genetic resources. Take urgent measures to put an end to poaching and trafficking of protected species of flora and fauna. Introduce measures to prevent the introduction of invasive alien species into terrestrial ecosystems. Mobilize and significantly increase financial resources from all sources to conserve and sustainably use biodiversity and ecosystems. Enhance global support for efforts to combat poaching and trafficking of protected species, including by increasing the capacity of local communities to pursue sustainable livelihood opportunities. The COVID-19 outbreak highlights the need to address threats to ecosystems and wildlife. In 2016, the United Nations Environment Program (UNEP) flagged a worldwide increase in zoonotic epidemics as an issue of concern. Specifically, it pointed out that 75 per cent of all emerging infectious diseases in humans are zoonotic and that these zoonotic diseases are closely interlinked with the health of ecosystems. IoT Application Solutions [214,215,216,217,218,219]: Forestry and agricultural sustainability, and taking care of the natural environment in general, is an essential field of work for solutions with IoT applications and sensors for monitoring, control, and supervision. The control of forest species has a great ally in IoT devices in drones, which through thermal camera technology, along with color, height, and forest mass detection cameras, can monitor large territories and help prevent damage. Large carbon sinks, such as forests and plant species, are essential for the health of people and ecosystems. Likewise, in agricultural management and forestry, advanced technological systems can control poaching and illegal practices, by means of alarm systems, video surveillance, detection, etc. Invasive species can often be detected in time, before they cause significant damage, by means of technological systems for controlling forest areas, with sensor devices for color, temperature, radiation, etc.
**Goal 16: Promote just, peaceful, and inclusive societies.** 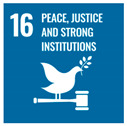 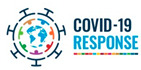	Targets 16.1–10, 16.a–b: Reduce all forms of violence and related mortality rates worldwide. End abuse, exploitation, trafficking, and all forms of violence and torture against children. Promote the rule of law and ensure equal access to justice for all. Reduce corruption and bribery in all its forms. Create effective and transparent institutions at all levels. Broaden and strengthen the participation of developing countries in global governance institutions. Provide access to a legal identity for all, in particular through birth registration. Ensure public access to information and protect freedoms. Promote and implement non-discriminatory laws and policies for sustainable development. Human rights put people center-stage. Responses that are shaped by and respect human rights result in better outcomes in beating the pandemic, ensuring healthcare for everyone and preserving human dignity. The UN Secretary General urged governments to be transparent, responsive, and accountable in their COVID-19 response and ensure that any emergency measures are legal, proportionate, necessary, and non-discriminatory. IoT Application Solutions [80,153,220,221,222,223,224,225]: We ask ourselves how technology and intelligent systems for data management, information control, and IoT can support and improve peace and justice in the world, to improve society, people’s health, and the environment. We believe that good technological planning is necessary, at all social, economic, and institutional levels, among others, to achieve a more just, peaceful, and egalitarian society. Technology reaches where other methods do not and can mean progress, which will bring peace and benefit communities. Violence can be prevented and mitigated with police control systems aided by technological devices, through data platforms and complex information systems that facilitate information sharing between different police institutions, at local, national, and international levels. Any form of terrorism or violence in society could be considerably reduced with more efficient and sophisticated management of crime data. The location systems that exist in any telephone device can decipher criminal activity movements, find kidnapped or missing persons, or help solve crimes, all by taking advantage of IoT technology, systems, and applications.
**Goal 17: Revitalize the global partnership for sustainable development.** 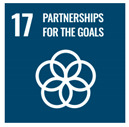 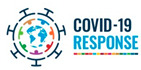	Targets 17.1–5: Finance. Ensuring that developed countries fulfill their official development assistance commitments. Most developing countries do not have sufficient domestic resources and fiscal space to fund adequate COVID-19 response and recovery measures. International cooperation and external finance are crucial. Targets 17.6–8: Technology. Improve cooperation in science, technology, and innovation. Promote the development of environmentally sound technologies and their transfer, dissemination, and diffusion to developing countries. Make fully operational the technology bank and the science, technology, and innovation capacity-building support mechanism for the least developed countries and increase the use of enabling technologies, in particular information and communications technology. Targets 17.9–12: Capacity building. Trade. Increase international support for effective and targeted capacity-building activities in developing countries to support national implementation plans for all SDGs. Promote a universal, open, non-discriminatory, and equitable multilateral trading system within the framework of the World Trade Organization. Targets 17.13–19: Regulatory and institutional coherence. Partnerships. Accountability. Increase global macroeconomic stability. Improve policy coherence for sustainable development. Respect each country’s policy space and leadership in establishing and implementing policies for poverty eradication and sustainable development. Enhance the Global Partnership for Sustainable Development, complemented by partnerships that mobilize and exchange knowledge, expertise, technology, and financial resources. Develop indicators to measure progress in sustainable development and complement gross domestic product. The World Health Organization (WHO), the UN Foundation and partners launched a first-of-its-kind Solidarity Response Fund to allow corporations and individuals to directly contribute to the WHO’s COVID-19 response. IoT Application Solutions [178,179,226,227,228,229]: Actions vary greatly among countries and territories, so institutional and regulatory coherence must be fostered for the improvement of society and its environment. Technology can help in this, through the introduction of policies to promote sustainable development and regulatory and institutional education. In the field of finance and transparency in economic transactions, IoT technology and applications can provide data management and control through advanced software systems and artificial intelligence. Intelligent systems and their multiple applications will be key to improving cooperation in science, technology, and innovation for the rational promotion of technologies and their dissemination in developing countries. In recent months, technology banks for the management of vaccine production, population control, movement of people in territories, etc., have been key to address the COVID-19 pandemic. Effective capacity building will require adequate support of IoT technology and applications, which are fundamental for proper management and control of operations. Universal, open, and equitable trade will rely on technological applications as an essential element for its optimal development. Localized and isolated commerce, for example in rural populations, will be able to take off in the national or international market through advanced communication and data management technology systems. This will be key to strategic trade management in all areas. Technological alliances will be a breakthrough for the achievement of the SDGs globally; interaction between territories will be rely on smart systems as part of their knowledge and resources. The management of data, statistics, and indicators in all sectors of a country is key to progress. In recent decades, indicators have been fundamental for the adoption of policies, actions, and strategies in the areas of health, population, economy, sustainable development, forest conservation, etc.

**Table 6:** Merits and demerits of SDGs and Application to IoT systems.

SDG	Main Goal	Merits	Demerits	References
1	No Poverty	IoT applications will be used to reduce poverty.	Mechanisms and protocols should be used to make patents, codes, and smart solutions more accessible.	[89,129,130,131,132,133]
2	Zero Hunger	Technology is key to food safety control and improved productivity.	The structures for supervision and provision of advanced IOT systems in food industries are still very scarce.	[3,134,135,136,137,138]
3	Health	There are many IoT developments, patents, and healthcare solutions that will benefit healthcare systems.	There is a lack of financial resources and greater multidisciplinarity and contacts between health governance and technological research centers.	[77,78,139,140,141,142,143,144,145,146,147]
4	Education	Increased quality education integrates smart systems and IoT applications.	There are still many shortcomings in telecommunications networks and quality interconnections so that technological applications can reach the entire population.	[148,149,150,151,152]
5	Gender Equality	Women’s safety from violence can be solved through IoT security systems.	Some devices are expensive, ineffective, or law enforcement does not have the training or human resources to monitor them.	[120,153,154,155]
6	Water and Sanitation	Water loss control and prevention is an essential field of work for IoT applications, in addition to drinking water control.	IoT applications are still largely unknown by small users of water and sanitation services.	[116,156,157,158]
7	Energy	IoT Energy is the present and future of energy systems. From generation to the end consumer, the technology improves the quality of energy.	Smart systems are mainly used by energy companies. Small consumers still do not know or do not have access to smart devices to control and save energy in their environment.	[159,160,161,162,163,164,165,166,167]
8	Economic Growth	Jobs and economic growth have a great ally with technology, which will improve and increase the specialization and training of people.	There is still a digital gap in the population that must be solved through dissemination, research, and academic training at all educational levels.	[25,131,168,169,170,171]
9	Infrastructure	Infrastructure and digitization of buildings and indoor and outdoor facilities is starting a revolution through IoT systems.	Much remains to be done in the training of professionals in the sector, so that they include in their projects and designs, IoT applications, and systems that will improve constructions.	[69,131,172,173,174,175,176,177]
10	Inequality	Inequality between territories can decrease if action plans that have technology at their core are included.	Costs must be reduced and a global plan for the integration of technological systems for the most disadvantaged communities must be implemented.	[131,171,178,179,180,181,182,183,184,185]
11	Cities	Smart Cities are already changing our living environment. Urban or rural territories are adapting to new times with IoT.	Security is a major issue in the management and control of data that are essential for a good implementation of IoT in different city systems.	[15,82,131,186,187,188,189,190,191,192,193,194,195]
12	Sustainable Production	Sustainability is a cross-cutting area that exists in all areas. IoT applications and data control mean better management of waste and production.	Security is a major issue in the management and control of data that are essential for a good implementation of IoT in different city systems.	[131,196,197,198,199,200,201,202,203]
13	Climate Change	Climate change has a great ally with IoT, for the control of glaciers, atmospheric pollution, transportation, etc.	It is taking too long to implement IoT systems. Time is against us.	[86,166,204,205,206,207,208,209]
14	Oceans	IoT can be used to control tides, tsunamis, marine pollution, renewable energies, fishing, etc.	There are problems in the costs of submerged systems, and the sea is still a very complex environment for accurate control.	[210,211,212,213]
15	Biodiversity	Forests and natural territories can be improved with technology and non-invasive surveillance.	Data management is a problem on many occasions, since a large number of monitoring elements are generated that require artificial intelligence for their improvement, which is sometimes a problem.	[214,215,216,217,218,219]
16	Peace, justice.	Peace and justice are often determined by accurate knowledge of conflict situations. With IoT security systems, this problem can be improved.	Technology can also be a weapon of war. Its use in the wrong hands can give terrorist groups a tool to commit attacks and bring suffering to society.	[80,153,220,221,222,223,224,225]
17	Partnership	IoT systems and their architectures and designs need alliances between countries to share patents, manufacturing materials, etc.	The ownership of some materials, such as rare earths, can lead to geo-strategic problems based on technology.	[178,179,226,227,228,229]
